# Adeno-associated Virus (AAV) Serotypes Have Distinctive Interactions with Domains of the Cellular AAV Receptor

**DOI:** 10.1128/JVI.00391-17

**Published:** 2017-08-24

**Authors:** Sirika Pillay, Wei Zou, Fang Cheng, Andreas S. Puschnik, Nancy L. Meyer, Safder S. Ganaie, Xuefeng Deng, Jonathan E. Wosen, Omar Davulcu, Ziying Yan, John F. Engelhardt, Kevin E. Brown, Michael S. Chapman, Jianming Qiu, Jan E. Carette

**Affiliations:** a Department of Microbiology and Immunology, Stanford University School of Medicine, Stanford, California, USA; b Department of Microbiology, Molecular Genetics and Immunology, University of Kansas Medical Center, Kansas City, Kansas, USA; c Department of Biochemistry and Molecular Biology, School of Medicine, Oregon Health & Science University, Portland, Oregon, USA; d Department of Anatomy and Cell Biology, University of Iowa, Iowa City, Iowa, USA; e Virus Reference Department, Public Health England, Colindale, London, United Kingdom; International Centre for Genetic Engineering and Biotechnology

**Keywords:** AAVR, adeno-associated virus, gene therapy, receptor-ligand interaction, viral receptor, virus overlay assay, virus-host interactions

## Abstract

Adeno-associated virus (AAV) entry is determined by its interactions with specific surface glycans and a proteinaceous receptor(s). Adeno-associated virus receptor (AAVR) (also named KIAA0319L) is an essential cellular receptor required for the transduction of vectors derived from multiple AAV serotypes, including the evolutionarily distant serotypes AAV2 and AAV5. Here, we further biochemically characterize the AAV-AAVR interaction and define the domains within the ectodomain of AAVR that facilitate this interaction. By using a virus overlay assay, it was previously shown that the major AAV2 binding protein in membrane preparations of human cells corresponds to a glycoprotein with a molecular mass of 150 kDa. By establishing a purification procedure, performing further protein separation by two-dimensional electrophoresis, and utilizing mass spectrometry, we now show that this glycoprotein is identical to AAVR. While we find that AAVR is an N-linked glycosylated protein, this glycosylation is not a strict requirement for AAV2 binding or functional transduction. Using a combination of genetic complementation with deletion constructs and virus overlay assays with individual domains, we find that AAV2 functionally interacts predominantly with the second Ig-like polycystic kidney disease (PKD) repeat domain (PKD2) present in the ectodomain of AAVR. In contrast, AAV5 interacts primarily through the first, most membrane-distal, PKD domain (PKD1) of AAVR to promote transduction. Furthermore, other AAV serotypes, including AAV1 and -8, require a combination of PKD1 and PKD2 for optimal transduction. These results suggest that despite their shared dependence on AAVR as a critical entry receptor, different AAV serotypes have evolved distinctive interactions with the same receptor.

**IMPORTANCE** Over the past decade, AAV vectors have emerged as leading gene delivery tools for therapeutic applications and biomedical research. However, fundamental aspects of the AAV life cycle, including how AAV interacts with host cellular factors to facilitate infection, are only partly understood. In particular, AAV receptors contribute significantly to AAV vector transduction efficiency and tropism. The recently identified AAV receptor (AAVR) is a key host receptor for multiple serotypes, including the most studied serotype, AAV2. AAVR binds directly to AAV2 particles and is rate limiting for viral transduction. Defining the AAV-AAVR interface in more detail is important to understand how AAV engages with its cellular receptor and how the receptor facilitates the entry process. Here, we further define AAV-AAVR interactions, genetically and biochemically, and show that different AAV serotypes have discrete interactions with the Ig-like PKD domains of AAVR. These findings reveal an unexpected divergence of AAVR engagement within these parvoviruses.

## INTRODUCTION

Adeno-associated viruses (AAVs) are nonenveloped, single-stranded DNA viruses of the *Dependoparvovirus* genus of the Parvoviridae family (1). Unlike most viruses, AAVs are innately nonpathogenic, poorly immunogenic, and broadly tropic, making them attractive gene delivery candidates for virus-based gene therapies (2). Despite the extensive utility of AAV vectors in several ongoing clinical trials and preclinical studies for severe, monogenic disorders (2–5), we understand little about how AAV initiates infection and penetrates the cell barrier (6). Additionally, it is unclear how this might differ among AAV serotypes, many of which display significant differences in transduction efficiency and tissue tropism (7, 8).

Most naturally occurring AAVs utilize glycan moieties for initial attachment to the cell surface, and these interactions have been well characterized for a number of serotypes (9). The interacting glycan moieties identified to date include heparan sulfate proteoglycans for AAV serotype 2 (AAV2), AAV3, and AAV6 (10); N-terminal galactose for AAV9 (11, 12); and specific N- or O-linked sialic acid moieties for AAV1, -4, -5, and -6 (9, 13–16). Postattachment, AAV is thought to engage a proteinaceous receptor(s) to mediate cellular entry, although less is known about these interactions. For AAV2, the most well-studied AAV serotype, several studies have investigated possible cellular receptors. Mizukami et al. (17) were the first to report characteristics of a putative AAV2 receptor, describing a 150-kDa glycoprotein detected in membrane fractions of AAV-permissive cells, which bound AAV2 particles in a virus overlay assay. Following this work, several proteinaceous coreceptors, such as fibroblast growth factor receptor 1 (FGFR-1) (18), integrin αVβ5 (19), and the hepatocyte growth factor receptor (c-Met) (20), were implicated in AAV2 entry. However, clustered regularly interspaced short palindromic repeat (CRISPR)-mediated knockout of FGFR-1 and c-Met in several cell lines did not substantially affect AAV2 transduction (21), suggesting that these coreceptors have accessory rather than essential roles in AAV2 transduction.

We recently identified the AAV receptor (AAVR) (also known as KIAA0319L) as an essential receptor for AAV transduction of human cells derived from a broad range of tissues and in an *in vivo* mouse model (21). Multiple serotypes, including AAV1, AAV2, AAV3B, AAV5, AAV6, AAV8, and AAV9, require AAVR for transduction. AAVR is a glycosylated membrane protein that is capable of recycling from the plasma membrane to the *trans*-Golgi network using the cellular endosomal network. The ectodomain of AAVR comprises of a MANEC (motif at the N terminus with eight cysteines) domain as well as five Ig-like domains known as PKD (polycystic kidney disease) domains (22). Ig-like domains have been associated with cell adhesion functions and are often exploited by viruses during cell entry (23). AAV also utilizes these Ig-like structures to facilitate transduction, specifically PKD domains 1 (PKD1) to PKD3 within the ectodomain of AAVR (21). Here, we show that AAVR is identical to the receptor previously implicated in the study by Mizukami et al. and further characterize the AAV-AAVR interaction. Our findings demonstrate that there are serotype-specific interactions with AAVR, which may suggest evolutionary differences in receptor usage between serotypes.

## RESULTS

### The major AAV2 binding protein in membrane preparations is identical to AAVR.

**FIG 1:**
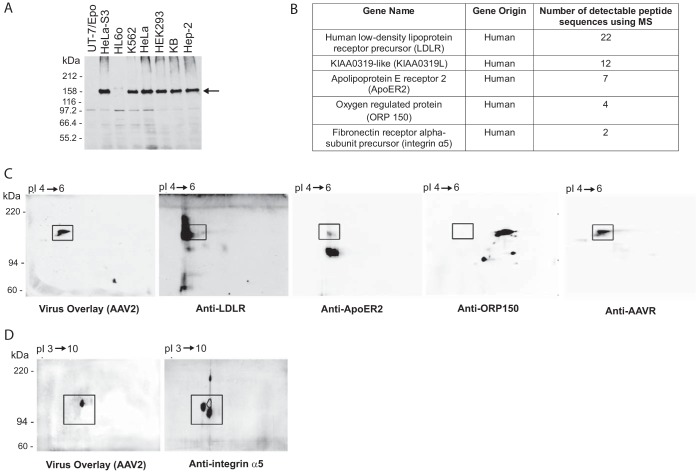
The identity of the AAV-BP is AAVR, the multiserotype AAV receptor. (A) Virus overlay assay of cell membrane fractions from different types of cells. Membrane proteins were extracted from various types of cells, and 100 μg of membrane proteins was used to perform a virus overlay assay with purified wild-type AAV2 particles (56). The arrow indicates a strong binding band at 150 kDa, designated AAV-BP. (B) Summary of the top five genes that correspond to the peptide sequences from the mass spectrometry analysis of the AAV-BP band. AAVR is also denoted KIAA0319L. (C) One hundred micrograms of PSA-purified HeLa S3 membrane proteins was separated on a 2-D gel and transferred onto a PVDF membrane for a virus overlay assay with rAAV2, followed by reprobing with anti-LDLR, anti-ApoER2, anti-ORP150, or anti-AAVR antibody. (D) One hundred micrograms of N- and O-deglycosylated crude HeLa S3 cell membrane proteins was separated by 2-D electrophoresis and underwent a virus overlay assay, followed by reprobing with a rabbit polyclonal antibody to integrin α5. Squares indicate an identical area of the membrane.

**FIG 2:**
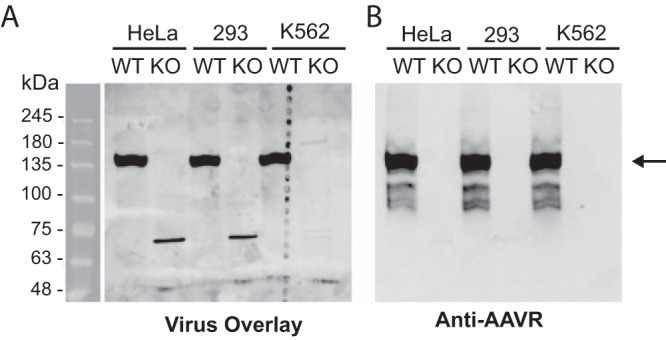
AAV-BP is absent in AAVR^KO^ cells, reiterating its identity as AAVR. Purified membrane proteins from wild-type (WT) and AAVR^KO^ (KO) cells (derived from the respective cell lines) were solubilized in PB–2% DMT. Twenty micrograms of the solubilized membrane proteins was separated on an SDS–6% PAGE gel and transferred onto a PVDF membrane for a virus overlay assay with rAAV2 (A), followed by reprobing with anti-AAVR antibody (B). The arrow indicates a strong band at 150 kDa in both the virus overlay and the immunoblot.

It was previously found that AAV2 binds a 150-kDa cell membrane glycoprotein (which we call AAV binding protein [AAV-BP] here) in a virus overlay assay using ^35^S-labeled AAV2 (17). We confirmed this virus-cellular protein interaction utilizing a modified virus overlay assay that makes use of unlabeled AAV2 and a monoclonal antibody (A20) against the intact AAV2 capsid. Cell membrane fractions taken from immortalized cell lines derived from a variety of human tissues were isolated and probed in this assay (Fig. 1A). The 150-kDa AAV-BP was detected strongly in membrane fractions of AAV2-permissive cells (HeLa S3, K562, HeLa, HEK293, KB, and Hep-2 cells) but was weakly detected or undetectable in cell lines known to be less permissive to AAV2 transduction (HL60 and UT7/Epo cells) (17, 21, 24). We went on to purify AAV-BP from the cell membrane fraction of HeLa cells, optimizing the purification process by using Pisum sativum agglutinin (PSA) lectin-coated beads and then jacalin-conjugated beads, which bound AAV-BP well. Upon mass spectrometry (MS) analysis of the protein excised from the gel in a region corresponding to where AAV binding activity was detected, we identified peptide sequences from a number of proteins, including the low-density lipoprotein receptor precursor (LDLR), apolipoprotein E receptor 2 (ApoER2), AAVR (KIAA0319L), oxygen-regulated protein 150 (ORP150), and integrin α5 (Fig. 1B). By using specific antibodies against each of the proteins identified, only those against AAVR precisely overlapped the region that displayed AAV2 binding activity in the virus overlay assay (Fig. 1C and D). These results indicate that the protein with the strongest binding activity in a virus overlay assay (AAV-BP) is identical to AAVR, the multiserotype receptor identified in an unbiased genetic screen for AAV2 transduction (21). This was further validated by an AAV2 virus overlay assay using isogenic knockout cells lines (created by using CRISPR-mediated genome editing), where the 150-kDa band of AAV-BP was detectable in wild-type (WT) but not in AAVR knockout (AAVR^KO^) cells (Fig. 2A and B).

### AAVR glycosylation is not essential for AAV2-AAVR interactions or AAV2 transduction.

**FIG 3:**
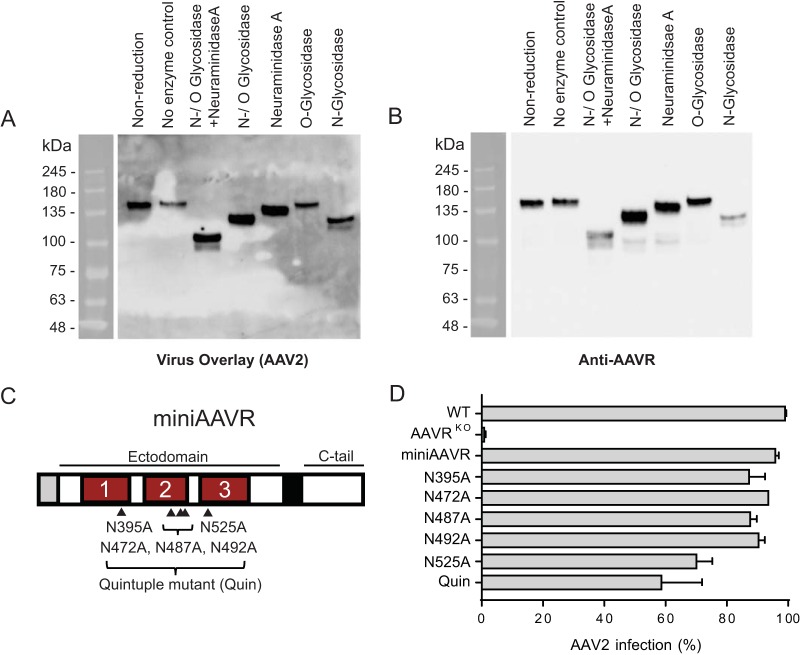
AAVR glycosylation is not crucial for its interaction with AAV2. (A and B) Purified HeLa S3 membrane proteins were solubilized in PB–2% DMT. Twenty micrograms of the solubilized membrane proteins was treated with various N- and O-deglycosylation enzymes prior to undergoing gel electrophoresis (SDS–6% PAGE), which was followed by a virus overlay assay with rAAV (A) and then reprobing with an anti-AAVR antibody (B). (C) Schematic depicting miniAAVR (comprising only PKD domains 1 to 3 in its ectodomain) and its 5 N-glycosylation sites, which were mutated from asparagine (N) to alanine (A) to create 6 glycosylation mutants, including Quin (carries mutations in all five sites). (D) scAAV2-CMV-RFP transduction of HeLa AAVR^KO^ cells stably expressing AAVR glycosylation mutants depicted in panel C (MOI of 20,000 vg/cell). Data in panel D depict means with standard deviations from triplicate transductions, where transgene expression was measured after 48 h.

AAVR has a predicted protein molecular mass of 108 kDa, but it is detectable at 150 kDa after immunoblotting (21), likely due to the effect of N- and O-linked glycosylation on protein migration during gel electrophoresis. To assess the importance of AAVR glycosylation on its interaction with AAV2, we treated cellular membrane preparations with deglycosylation enzymes (Fig. 3A and B). *N*-Glycosidase treatment led to a shift in migration, implying that AAVR is an N-linked glycosylated protein. Concomitant treatment with N- and O-linked glycosidases and neuraminidase (both of the latter two enzymes are required to cleave O-linked disaccharides) led to a further shift, suggesting that AAVR is also an O-linked glycosylated protein. None of the deglycosylation treatments reduced the binding of AAV2 in a virus overlay assay (Fig. 3A), demonstrating that glycosylation is not required for binding. To test the effect of N-linked glycosylation in a functional transduction assay, we used the stable expression of AAVR mutants in HeLa AAVR^KO^ cells. We have previously shown that the expression of an AAVR minimal mutant (miniAAVR), consisting of a signal peptide, PKD domains 1 to 3 of the ectodomain, the transmembrane domain, and the C-terminal cytoplasmic region, is able to efficiently rescue AAV2 transduction in AAVR^KO^ cells (21). This indicates that these PKD domains are sufficient for binding AAV2 and mediating entry. Within the ectodomain of miniAAVR, there are 5 putative asparagine glycosylation sites (indicated in Fig. 3C), which were mutated to alanine (individually or in combination) to prevent N-linked glycosylation. Mutation N525A and the quintuple mutant mildly affected AAV2 transduction, whereas the other mutations did not appreciably affect the transduction efficiency compared to that of the wild-type construct (Fig. 3D). Thus, by this cellular assay, N-linked glycosylation of AAVR facilitates optimal AAV2 transduction but is not strictly required.

### AAVR PKD2 is critical for the interaction of AAV2 with AAVR.

**FIG 4:**
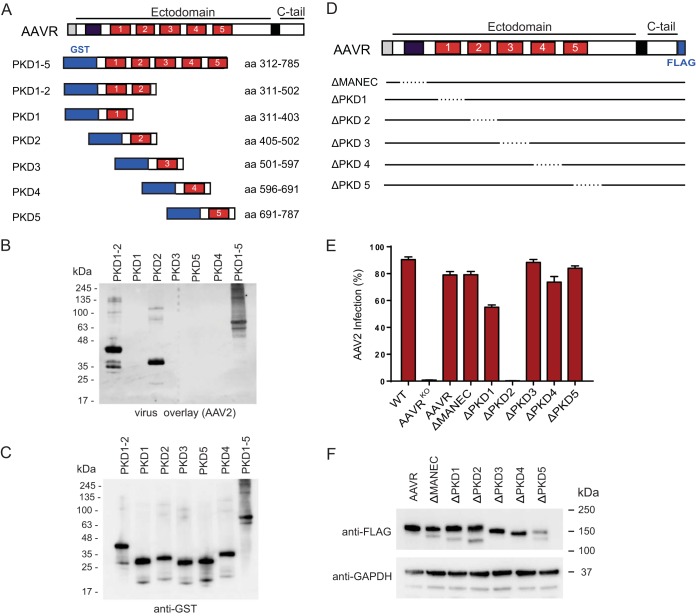
AAV2 interacts with the second PKD domain in AAVR's ectodomain, and this interaction is critical for transduction. (A) Schematic depicting AAVR domains and various GST-tagged AAVR ectodomain constructs expressed in E. coli. (B and C) Gel electrophoresis (SDS–12% PAGE) was carried out on bacterial lysates of equal volumes of E. coli cells transformed with the respective constructs, and a virus overlay assay with rAAV2(Luc/mCherry) was performed (B), followed by reprobing with an anti-GST antibody (C). (D) Schematic depicting AAVR domains and deletion mutants, which remove a single domain per mutant. (E) scAAV2-CMV-RFP transduction of HeLa AAVR^KO^ cells stably expressing AAVR deletion mutants depicted in panel D (MOI of 20,000 vg/cell). (F) Immunoblot of AAVR deletion mutants depicted in panel D, using anti-FLAG antibody and anti-GAPDH antibody. Cell pellets of 1 × 10^6^ cells were lysed with Laemmli SDS sample buffer containing 5% β-mercaptoethanol and were separated by SDS–4 to 15% PAGE, followed by immunoblotting. Data in panel E depict means with standard deviations from triplicate transductions, where transgene expression was measured after 48 h.

We previously identified AAVR PKD domains 1 to 3 to include the binding domain for AAV2 (21). To further characterize the AAVR-AAV2-interacting region, we created glutathione *S*-transferase (GST)-tagged AAVR ectodomain mutants consisting of individual PKD domains or combinations of sequential domains (Fig. 4A). We expressed the respective mutants in Escherichia coli and performed a virus overlay assay, where we observed binding of AAV2 to PKD domains 1 to 5, PKD domains 1 and 2, and PKD domain 2 alone (Fig. 4B and C). In addition, AAV2 transduction of AAVR^KO^ cells stably expressing AAVR deletion mutants consisting of individual domain deletions (Fig. 4D and F) demonstrated that only the deletion of PKD2 completely abrogated AAV2 transduction (Fig. 4E). The AAVR deletion mutant missing PKD1 showed a less pronounced decrease in transduction efficiency. These data indicate that PKD2 is the critical region for AAV2 binding and transduction, with PKD1 playing a possible accessory role.

**FIG 5:**
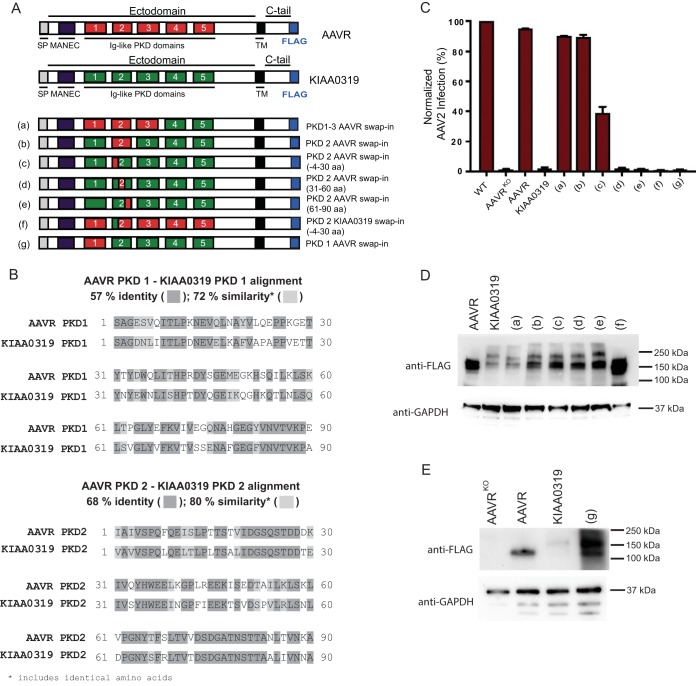
Domain swapping between AAVR and its homologue, KIAA0319, shows that PKD2 is important for AAV2. (A) Schematic depicting domains of AAVR and KIAA0319 and swap mutants that have ectodomain regions that have been swapped from AAVR into KIAA0319 (mutants a to e and g) or from KIAA0319 to AAVR (mutant f). SP, signal peptide; TM, transmembrane. (B) Amino acid sequence alignment between AAVR PKD1 and KIAA0319 PKD1 and between AAVR PKD2 and KIAA0319 PKD2. Percent identity indicates how many amino acids are exactly the same; similarity compares the overall sequences and determines their likeness, taking into account amino acid identity and charge. (C) scAAV2-CMV-RFP transduction of the respective swap mutants depicted in panel A (MOI of 100,000 vg/cell), where transductions of mutants were normalized to the transduction of the WT. (D and E) Immunoblotting of swap mutants depicted in panel A, using anti-FLAG antibody or anti-GAPDH antibody. Cell pellets of 1 × 10^6^ cells were lysed with Laemmli SDS sample buffer and were separated by SDS–4 to 15% PAGE, followed by immunoblotting. Data in panel C depict means with standard deviations from triplicate transductions, where transgene expression was measured after 48 h.

**FIG 6:**
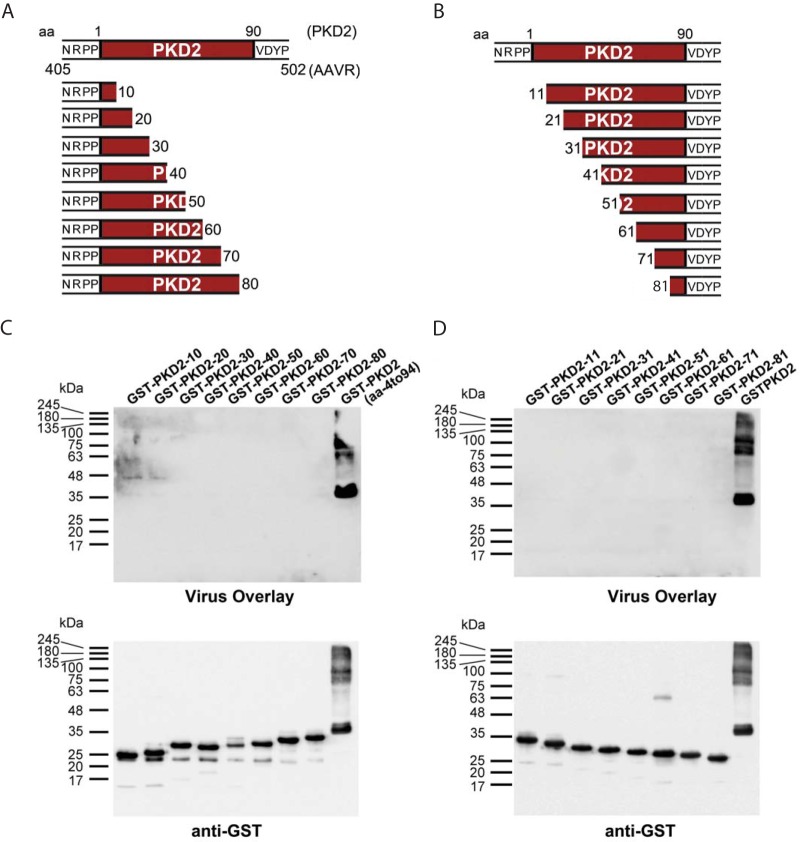
Binding of AAVR PKD2 in virus overlay assays requires full-length AAVR PKD2. (A and B) Schematic depicting GST-tagged AAVR ectodomain constructs comprising PKD2 and various PKD2 ectodomain mutants, created by removing C-terminal amino acids (A) or N-terminal amino acids (B). (C and D) Gel electrophoresis was carried out on bacterial lysates transformed with the respective constructs in panels A and B, and a virus overlay assay with AAV2 was performed, followed by reprobing with an anti-GST antibody for N-terminal deletion constructs (C) or C-terminal deletion constructs (D).

To further define the region of PKD2 necessary for AAV2 transduction, we constructed domain swap mutants in the AAVR homologue, KIAA0319, which shares approximately 60% amino acid sequence similarity with AAVR. AAVR is known to be KIAA0319-like because of its structural likeness to KIAA0319 (Fig. 5A). The two proteins both comprise a MANEC domain along with 5 PKD domains in their ectodomain and are both glycosylated, type I transmembrane proteins (25). Moreover, PKD1 and PKD2 of KIAA0319 share 72% and 80% similarities, respectively, with the corresponding PKD domains in AAVR (Fig. 5B). Importantly, despite its similarity to AAVR, KIAA0319 cannot be utilized by AAV2 to successfully transduce cells (Fig. 5C). Thus, we utilized KIAA0319 as a backbone in which regions of KIAA0319 were replaced with AAVR (Fig. 5A, D, and E). We then stably expressed these mutants in AAVR^KO^ cells and assessed AAV2 transduction. A swap-in of AAVR PKD domains 1 to 3 into KIAA0319 (mutant a) and PKD2 alone (mutant b) fully rescued AAV2 transduction, whereas a swap-in of PKD1 alone (mutant g) did not rescue transduction (Fig. 5C). This is consistent with the binding and deletion mutant data and reinforces the major role of AAVR PKD2 for AAV2. Additionally, a swap-in of only an N-terminal region of AAVR PKD2 (∼30 amino acids [aa]) partially rescued transduction, indicating that this region is particularly important for AAV2 transduction (mutant c). Reiterating this point, swapping of the same region of KIAA0319 into AAVR (mutant f) eliminated the capacity of AAVR to rescue AAV2 transduction. Swapping of the C-terminal region of AAVR PKD2 into KIAA0319 (mutants d and e) did not rescue transduction. Using a collection of deletion constructs of PKD2, we observed that none of the mutants, even the ones containing small N- and C-terminal PKD2 deletions (Fig. 6A and B), were able to bind to AAV2 in a virus overlay assay (Fig. 6C and D). Thus, N-terminal of AAVR PKD2 is able to partially rescue AAV2 transduction in the domain swaps yet is not able to bind AAV2 particles in the *in vitro* binding assay. This suggests that full PKD2 of AAVR is required for optimal binding and transduction.

### AAV5 requires AAVR PKD1 for binding and functional transduction.

**FIG 7:**
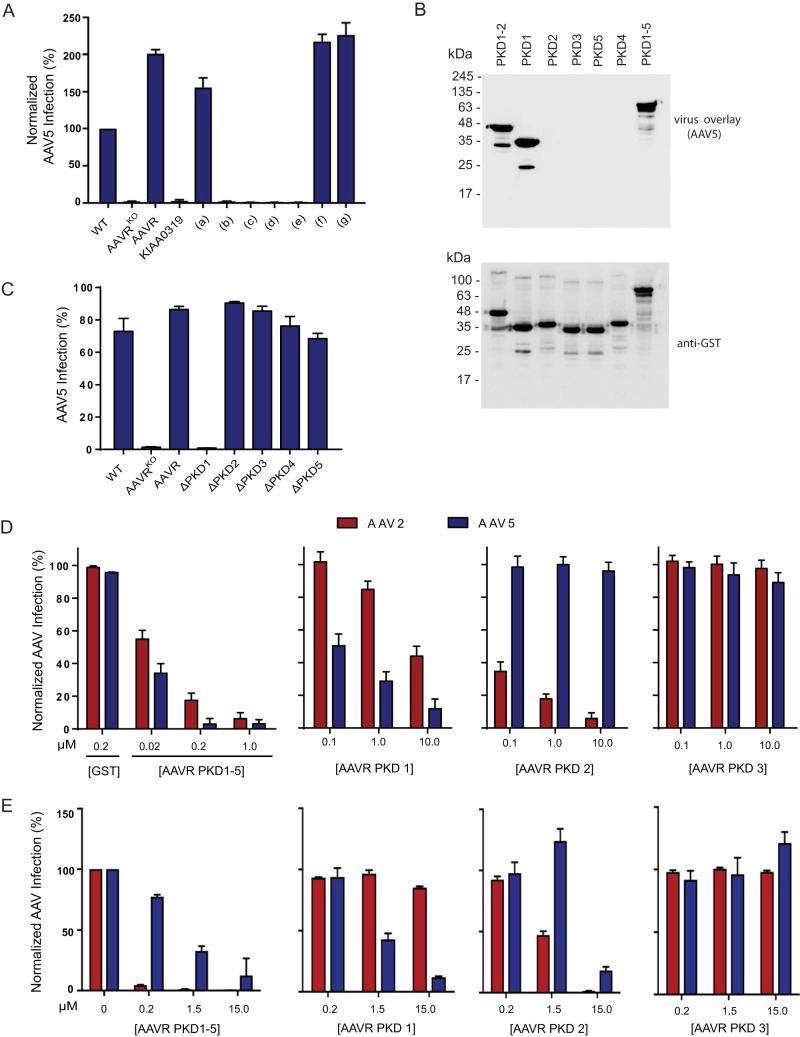
AAV5 has evolved to interact with a different AAVR PKD domain than AAV2. (A) scAAV5-CMV-GFP transduction of the respective swap mutants depicted in Fig. 5A (MOI of 100,000 vg/cell), where transductions of mutants were normalized to the transduction of WT cells. (B) Gel electrophoresis was carried out on bacterial lysates transformed with the respective constructs from Fig. 4A, and a virus overlay assay with rAAV5(Luc/mCherry) was performed, followed by reprobing with an anti-GST antibody. (C) scAAV5-CMV-GFP transduction of HeLa AAVR^KO^ cells stably expressing AAVR deletion mutants depicted in Fig. 4D (MOI of 100,000 vg/cell). (D) Virus neutralization assay whereby HeLa cells were incubated with different concentrations of the respective soluble AAVR variants or GST during rAAV2(Luc/mCherry) or rAAV5(Luc/mCherry) transduction (MOI of 7,500 vg/cell). Data depict means with standard deviations from triplicate transductions, where transgene (luciferase) expression was measured after 48 h. (E) Independent virus neutralization assay using AAVR PKD ectodomains. HeLa cells were incubated with different concentrations of the respective soluble AAVR variants during scAAV2-CMV-GFP or scAAV5-CMV-GFP transduction (MOI of 12,000 vg/cell). Data depicted in panels D and E represent normalized means (relative to cells incubated with PBS) with standard deviations from triplicate transductions, where transgene expression was measured after 24 h by using flow cytometry.

Our previous study demonstrated that AAVR is an essential receptor for multiple AAV serotypes (21), including AAV5, which is evolutionarily distant from AAV2 and shows only 57% identity in the capsid amino acid sequence (26–28). To explore the hypothesis that the interaction interfaces of different AAV serotypes with AAVR are distinct, we used the collection of domain swap mutants and probed AAV5 transduction. In stark contrast to the results obtained with AAV2, neither full-length PKD2 (mutant b) nor the N-terminal mutant (mutant c) rescued AAV5 transduction when swapped into KIAA0319 (Fig. 7A). Additionally, the AAVR mutant with a partial PKD2 swap of KIAA0319 (mutant f), which completely lost its ability to facilitate AAV2 transduction, was fully competent in mediating AAV5 transduction. Interestingly, when the most membrane-distal PKD domain, PKD1, was swapped into KIAA0319 (mutant g), we observed a rescue of transduction to the same levels as those with full-length AAVR, suggesting that AAV5 evolved to require PKD1 rather than PKD2 for functional transduction (Fig. 7A). In agreement with this, an AAV5 virus overlay assay using individual AAVR PKD ectodomain mutants demonstrated that AAV5 bound specifically to AAVR PKD1 and not PKD2 (Fig. 7B). Consistent with data from the virus overlay assay and swap experiments, AAV5 transduction of AAVR^KO^ cells stably expressing AAVR single deletion mutants confirmed the strict requirement of AAVR PKD1 for AAV5 transduction (Fig. 7C). To test the differential requirements for PKD domain usage further, we used a virus transduction assay, where increasing amounts of soluble variants of AAVR domains were incubated with cells during AAV2 or AAV5 transduction. The transduction of both AAV2 and AAV5 was inhibited by increasing amounts of PKD1-5, while the addition of PKD3 had no effect on either virus (Fig. 7D). AAV2 transduction was potently blocked by PKD2 and, to a lesser degree, by PKD1, in concurrence with the deletion mutant data (Fig. 4E). Conversely, AAV5 transduction was potently blocked by PKD1 and unaffected by the presence of PKD2 at low concentrations, in agreement with the transduction and virus overlay data (Fig. 7D). However, at a higher concentration of soluble PKD2, we observed a significant decrease in AAV5 transduction (Fig. 7E), suggesting that PKD2 may play a minor role in the AAV5-AAVR interaction. The functional relevance of this is unclear because the deletion of PKD2 does not reduce AAV5 transduction (Fig. 7C). Together, these results imply complexity in the interaction of AAVR with different serotypes, where AAV2 utilizes PKD2 and, to a lesser degree, PKD1 to enable vector transduction, whereas AAV5 primarily requires PKD1.

### Serotype-specific utilization of AAVR PKD domains 1 and 2.

**FIG 8:**
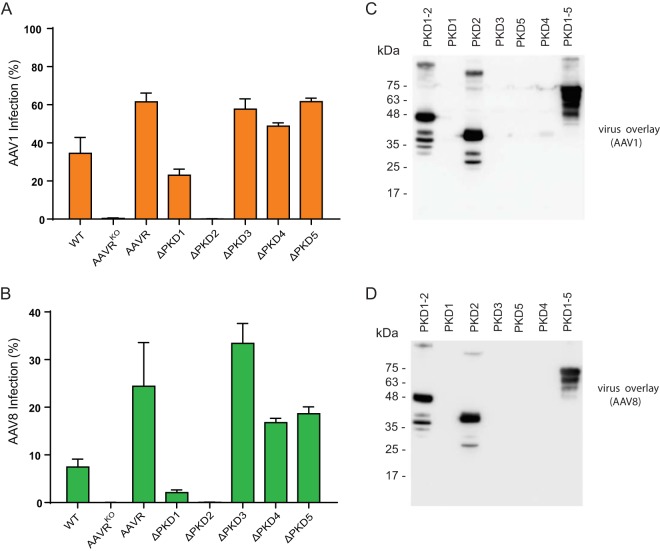
Utilization of AAVR PKD domains 1 and 2 by different AAV serotypes. (A and B) AAV1 (A) or AAV8 (B) transduction of HeLa AAVR^KO^ cells stably expressing AAVR deletion mutants depicted in Fig. 4D (MOI of 100,000 vg/cell). Data depict means with standard deviations from triplicate transductions, where transgene expression was measured after 48 h. (C and D) Gel electrophoresis (SDS–12% PAGE) was carried out on bacterial lysates of equal volumes of E. coli cells transformed with the respective constructs described in the legend of Fig. 4A, and a virus overlay assay with rAAV1(Luc/mCherry) (C) or rAAV8(Luc/mCherry) (D) was performed.

In view of the disparity in AAVR domain dependence between AAV2 and AAV5, we evaluated the transduction of AAV1 (Fig. 8A) and AAV8 (Fig. 8B) in AAVR^KO^ cells stably expressing AAVR single deletion mutants. Notably, AAV1 and AAV8 were similar to AAV2 in their critical requirement for PKD2, although they appeared to depend more strongly on PKD1 than AAV2 did for transduction. Both AAV1 and AAV8 bound to only PKD2 in a virus overlay assay (Fig. 8C and D), suggesting a higher affinity for PKD2 in this assay.

## DISCUSSION

A thorough understanding of how AAV interacts with its multiserotype receptor AAVR has the potential to lead to improvements in future vector design and modulations in tissue-targeting strategies. In this study, we establish that AAV-BP, the component of cell membrane extracts reported 2 decades ago to have the greatest AAV2 binding activity, is identical to the multiserotype receptor, AAVR. We further uncover that several different AAV serotypes have evolved distinct interactions for engaging the same receptor, AAVR, upon which they all have a strict dependence.

It has long been established that AAV2 attaches to cells via interactions with heparan sulfate proteoglycan (10). However, it was unclear if a proteinaceous receptor was a critical requirement for AAV2 infection. We recently identified AAVR in an unbiased genetic screening approach as being an essential receptor required for infection by AAV2 as well as by several other AAV serotypes (21). To further characterize AAVR, we revisited the first reported AAV receptor study (17) to determine if characteristics of the previously described AAV2 binding protein (AAV-BP) matched those of AAVR. AAV-BP, a highly glycosylated protein with a molecular mass of 150 kDa, was found to have dominant binding to AAV2 in cell membrane extracts from several human cell types. We show here, using mass spectrometry, modified virus overlay assays, and deglycosylation treatments, that AAVR is identical to the previously described AAV-BP. Moreover, using isogenic AAVR knockout cell lines, we unambiguously demonstrate that AAV-BP is present only in cells that express AAVR. This study thus provides an independent yet complementary approach to the identification of AAVR as an essential AAV2 receptor, demonstrating that the traditional virus overlay assay combined with a membrane protein purification procedure can still be a powerful approach to identify viral receptors. Notably, several other proteinaceous coreceptors for AAV2 have been described (18–20), but none scored in the genetic screens or are implicated in the current proteomic analysis of virus overlays.

Although the role of AAVR in cellular physiology is unclear, the structure indicates that it is a glycoprotein, containing Ig-like PKD domains in its ectodomain (21, 29). Many glycoproteins that contain repeats of Ig-like domains have been identified as viral receptors, including the human rhinovirus (HRV) major receptor, ICAM-1, and the coxsackievirus/adenovirus receptor, CAR (23). Here, we observed that the enzymatic removal of both O- and N-glycosylation decreased AAVR to a molecular mass of around 100 kDa, approximately the predicted molecular mass without modifications, which suggests that AAVR itself is not a heparan sulfate proteoglycan. We show that different AAV serotypes specifically interact with the Ig-like PKD domains of AAVR but demonstrate that glycosylation is not a prerequisite for AAV2 binding and transduction. This is similar to what has been noted for other nonenveloped viruses such as HRV and coxsackievirus, where the interactions between the virus and Ig-like domains in their receptors occur independently of N-linked glycosylation (30, 31). Interestingly, our glycosylation studies showed a slight reduction in functional transduction upon mutation of the Asp_525_ residue located in AAVR PKD3 as well as in the mutant carrying all 5 putative glycosylation sites. Because PKD3 is not critical for AAV2 transduction *per se* (Fig. 4E), we hypothesize that this observed reduction could be due to a disturbed folding or trafficking of AAVR, which potentially might require N-linked glycosylation at this site.

AAVR shares striking homology with KIAA0319, a candidate gene that may contribute to dyslexia (25, 32). Specifically, the ectodomain of KIAA0319 also contains 5 Ig-like PKD domains, which have an implied role in cell-to-cell adhesion (22, 33), and KIAA0319 is a highly glycosylated, transmembrane protein. Despite these similarities, AAV cannot utilize KIAA0319 instead of AAVR to gain entry into the cell. Thus, to determine which domain of AAVR is critical for transduction, we took advantage of the homology between AAVR and KIAA0319 and swapped regions of AAVR into KIAA0319, and vice versa, evaluating which mutants would rescue AAV transduction. Notably, we found that although AAVR is required for multiple serotypes, its interaction with AAV differs between serotypes. We show that AAV has evolved to interact specifically with the most membrane-distal domains (PKD1 and PKD2). In particular, AAV2 functionally interacts mainly with PKD2, with some contribution by PKD1, while AAV5 predominantly interacts with PKD1. It is interesting to note that, similarly to AAV, other viruses that engage receptors with Ig-like domains also utilize the most membrane-distal domain (34–39) as their contact site. For example, ICAM-1 contains 5 Ig-like domains (D1 to D5), of which the most membrane-distal domain (D1) directly interacts with HRV14, D2 contributes somewhat to binding, and D3 to D5 provide spacing to allow accessibility to the binding sites (38, 39).

The difference in AAVR interactions among the various serotypes is intriguing, as we have tested serotypes from different clades. AAV1 (clade A) and AAV2 (clade B) functionally interact mainly with PKD2, with some contribution by PKD1, while AAV5 (no designated clade) predominantly interacts with PKD1, and AAV8 (clade E) requires both PKD1 and -2 to transduce cells. These striking differences in AAV-AAVR functional interactions could be related to the origin from which the serotype was initially isolated, as AAV5 was isolated from human tissues (40, 41), while many other AAVs, including AAV1 and AAV2, were first found as contaminants in laboratory adenovirus stocks (42–46), and AAV8 was isolated from nonhuman primate samples (47). Additionally, AAV5 speciated early and is quite distinct from the evolutionary lineage to which the other AAV serotypes belong (28). As sequence diversity between AAV serotypes has allowed AAV to evolve footprints that preferentially interact with different glycan receptors, such as heparan sulfate and sialic acid (9), our results suggest that AAV serotypes also evolved distinct interactions with AAVR. This may or may not be related to their glycan dependence, something to be investigated in future studies for a more in-depth understanding of virus-receptor engagement. Moving forward, structural studies that complement the data presented here will further define the AAV-AAVR interaction domain in greater molecular detail and will be necessary to establish the exact binding sites, both in AAVR and on the viral capsids.

## MATERIALS AND METHODS

### Cell lines and primary cells.

All cell lines were grown in medium supplemented with 10% fetal calf serum (FCS) (Sigma, St. Louis, MO), 100 IU/ml penicillin-streptomycin (Sigma, St. Louis, MO), and 2 mM l-glutamine (Sigma, St. Louis, MO) and grown in a humidified incubator at 37°C with 5% CO_2_. KB, HeLa, HeLa S3, 293, Hep-2, K562, and HL60 cells were purchased from the American Type Culture Collection (ATCC) (Manassas, VA). HeLa S3 cells were cultured as a suspension in F-12 medium, and other cells were maintained as a monolayer in Dulbecco's modified Eagle's minimal medium (DMEM). UT7/Epo cells were provided by N. Komatsu (Jichi Medical School, Japan) and cultured in DMEM with the same supplements as those described above plus erythropoietin (EPO) at 1 U/ml. The isogenic AAVR knockout cell lines used in this study were generated by using CRISPR-Cas9 technology and were described previously (21). They were maintained in the same way as parental cell lines. The AAVR^KO^ cells referred to in the genetic experiments shown in Fig. 3 to 8 are HeLa AAVR^KO^ cells.

### Viruses and recombinant vectors.

WT AAV2 was propagated and purified by CsCl ultracentrifugation, performed as previously reported (48). The protein concentration of the virus preparation was measured by using the bicinchoninic acid (BCA) protein assay reagent (Pierce Co., Rockford, IL).

Recombinant AAV (rAAV) vectors carrying reporter genes, rAAV1(Luc/mCherry), rAAV2(Luc/mCherry), rAAV5(Luc/mCherry), or rAAV8(Luc/mCherry), were produced by cotransfecting 293 cells with pAVF5tg83luc-CMVmChery, an adenoviral helper plasmid (pHelper; Agilent, Santa Clara, CA), and pAAV2RepAV1Cap, pAAV2RepCap, pAAV2RepAV5Cap, or pAAV2RepAV8Cap. At 48 h posttransfection, rAAV vectors were purified by two consecutive rounds of CsCl ultracentrifugation as previously reported (49). The titers of rAAV vector stocks were estimated to be ∼5 × 10^12^ viral genomic copies (vgc)/ml by quantitative PCR. For the rAAV transduction assays with the rescued AAVR^KO^ cell lines, recombinant self-complementary reporter vectors scAAV2-CMV-RFP and scAAV5-CMV-GFP were used, which were purchased from the University of North Carolina, Chapel Hill, Gene Therapy Center Vector Core.

### Antibodies.

The following antibodies were used in this study: mouse polyclonal anti-KIAA0319L (catalogue number ab105385) from Abcam (Cambridge, CA); mouse monoclonal anti-FLAG antibody (catalogue number F3165) from Sigma-Aldrich (St. Louis, MO); mouse monoclonal anti-glyceraldehyde-3-phosphate dehydrogenase (GAPDH) antibody (catalogue number GT239) from Genetex (Irvine, CA); mouse monoclonal anti-GST antibody (catalogue number A00865) from GenScript (Piscataway, NJ); anti-intact AAV1 (clone ADK1a, catalogue number 03-651150), anti-intact AAV2 (clone A20, catalogue number 03-61055), anti-intact AAV5 (clone ADK5a, catalogue number 03-651148), and anti-intact AAV8/9 (clone ADK8/9, catalogue number 03-651161) antibodies from American Research Products, Inc. (Waltham, MA); anti-LDLR (catalogue number A304-417A) from Bethyl Laboratories, Inc. (Montgomery, TX); anti-ApoE2 (catalogue number NB100-2216) from Novus Biologicals (Littleton, CO); anti-ORP150 (catalogue number AF5558) from R&D Systems (Minneapolis, MN); and anti-integrin α5 antibody (catalogue number AB1921) from EMD Millipore (Billerica, MA). Antibodies were used at dilutions suggested by the manufacturers.

### Enzymatic deglycosylation of cell membrane proteins.

Peptide-*N*-glycosidase F (PNGase F) (catalogue number P0705S), *O*-glycosidase (catalogue number P0733S), and α2-3,6,8,9-neuraminidase A (catalogue number P0722S) were purchased from New England BioLabs (NEB) (Ipswich, MA). Eighteen micrograms of 2% *n*-dodecyl-β-d-maltoside (DMT)-solubilized HeLa S3 membrane proteins (1.4 mg/ml) was first incubated with 10× denaturing buffer in a volume of 20 μl at 100°C for 10 min. The reaction mixture was then incubated with GlycoBuffer 2 and *N*-glycosidase F (1,000 U); *O*-glycosidase (80,000 U); both *N*-glycosidase F (1,000 U) and *O*-glycosidase (80,000 U); or *N*-glycosidase F (1,000 U), *O*-glycosidase (80,000 U), and α2-3,6,8,9-neuraminidase A (80 U) at 37°C for 1 h in a volume of 40 μl. Mock digestion reaction mixtures were set up without the addition of any enzymes.

### Purification of AAV-BP and mass spectrometry.

Cell membrane proteins were prepared from 200 ml of HeLa S3 cell pellets as previously reported (17), solubilized with PB–2% DMT buffer (20 mM phosphate buffer [pH 7.2], 1 M NaCl, 1 mM CaCl_2_, 1 mM MgCl_2_, protease inhibitors cocktails, and 2% DMT), and loaded onto an XK 16/70 column (Pharmacia) containing ∼100 ml of PSA lectin-conjugated agarose (E-Y Lab, Inc., San Mateo, CA). After washing with PB–0.1% DMT, PSA binding proteins were eluted with elution buffer (100 mM methyl-α-d-glucopyranoside and 100 mM methyl-β-d-mannopyranoside in PB–0.1% DMT), followed by dialysis against TE–0.05% DMT buffer (20 mM Tris-HCl [pH 7.2], 1 mM ETDA, 0.05% DMT). NaCl was added to the dialyzed protein solution to 1 M and loaded onto a jacalin lectin-immobilized agarose column. After extensive washing with wash buffer (20 mM Tris-HCl [pH 7.2], 1 M NaCl, 1 mM ETDA, 0.05% DMT), the jacalin lectin binding protein was eluted with 0.4 M melibiose in wash buffer. The eluted protein was dialyzed against TE–0.01% DMT and concentrated by ultrafiltration with stirred cells (Amicon, Inc., Beverly, MA) to a concentration of 4 mg/ml. Approximately 10 mg of the lectin-purified protein was loaded onto a Prep Cell (model 491; Bio-Rad Laboratories, Hercules, CA) each time, followed by preparative electroelution. The fractions with AAV2 binding activity were combined and concentrated by ultrafiltration. The above-described concentrated protein preparation was further separated by isoelectric focusing (IEF) at pH 4 to 6, followed by a second sodium dodecyl sulfate (SDS)–6% polyacrylamide gel electrophoresis (PAGE) step. AAV-BP was located by performing a virus overlay assay. The protein spot with binding activity (at pI ∼4.5) was cut off and subjected to protein identification. Protein identification was performed at the Harvard Microchemistry Facility (Cambridge, MA) by microcapillary reverse high-performance liquid chromatography (HPLC)–nanoscale electrospray tandem mass spectrometry (μLC-MS/MS) on a Finnigan LCQ quadrupole ion trap mass spectrometer.

### Two-dimensional gel electrophoresis of cell membrane proteins.

Each aliquot of the sample (100 μl) was dialyzed overnight against 5 mM Tris-HCl (pH 6.8) using a 6,000- to 8,000-molecular-weight (MW)-cutoff (MWCO) membrane at 5°C. Each aliquot was lyophilized and redissolved in 50 μl of a 1:1 dilution of SDS boiling buffer-urea sample buffer before loading. Two-dimensional (2-D) electrophoresis was performed according to the carrier ampholyte method of isoelectric focusing (50, 51) by Kendrick Labs, Inc. (Madison, WI), as follows. Isoelectric focusing was carried out in a glass tube with an inner diameter of 2.3 mm using 2% pH 3 to 10 or pH 4 to 6 Servalytes (Serva, Heidelberg, Germany) for 9,600 V · h. One microgram of an IEF internal standard, tropomyosin, was added to the sample. This protein migrates as a doublet with a lower polypeptide spot at a MW of 33,000 and pI 5.2. The enclosed tube gel pH gradient plot for this set of Servalytes was determined with a surface pH electrode. After equilibration for 10 min in buffer “O” (10% glycerol, 50 mM dithiothreitol, 2.3% SDS, and 0.0625 M Tris [pH 6.8]), each tube gel was sealed to the top of a stacking gel, which was on top of an 8% acrylamide slab gel (0.75 mm thick) poured with lower-gel buffer at pH 8.0. SDS slab gel electrophoresis was carried out for about 10 h at 15 mA/gel. After slab gel electrophoresis, the gel was stained Coomassie brilliant blue R-250 or silver stained. The gels for blotting were placed into transfer buffer (10 mM *N*-cyclohexyl-3-aminopropanesulfonic acid [CAPS] [pH 11], 10% methanol [MeOH]) and transblotted onto a polyvinylidene difluoride (PVDF) membrane overnight at 200 mA and approximately 100 V per two gels.

### Plasmid construction. (i) pLenti-based mammalian expression constructs.

To generate all recombinant constructs for genetic experiments, a Gibson assembly reaction kit (New England BioLabs, UK) was used to insert the gene of interest into a lentivirus-based destination (DEST) vector, pLenti-CMV-Puro-DEST (w118-1) (Addgene plasmid 17452) digested with EcoRV to remove the DEST cassette (a gift from Eric Campeau) (52). AAVR and derived AAVR genes were amplified from AAVR-FLAG or miniAAVR, constructed as described previously (21). The KIAA0319 gene was amplified from a cDNA clone (GenBank accession number BC140821) (clone 9021689; GE Dharmacon, Lafayette, CO) and Gibson cloned into pLenti-CMV-Puro-DEST by using primers 5′-TGTGGTGGAATTCTGCAGATACCATGGCGCCCCCCACAG-3′ and 5′-CGGCCGCCACTGTGCTGGATTTACTTATCGTCGTCATCCTTGTAATCTCTGTCCTTTGAGCAATAACTG-3′, introducing a FLAG epitope in frame with the C-terminal end of KIA0319.

To generate the PKD deletion constructs of AAVR, two PCR products were generated for Gibson cloning. The extreme 5′-end primer (5′-GACTCTAGTCCAGTGTGGTG-3′) and 3′-end primer (5′-ATCCAGAGGTTGATTGTCGAG-3′) were similar for the constructs. Primers used to amplify the fragments for the constructs were 5′-CCATACCCAGTTATAAAGGAACTGCGGCCCCCCATTGCTATTG-3′ and 5′-CAGTTCCTTTATAACTGGGTATGG-3′ for ΔPKD1, 5′-GCCAGAGCCCCGTAAGCCCCCTGTGGCCAACG-3′ and 5′-CTTACGGGGCTCTGGC-3′ for ΔPKD2, 5′-GTGAACAAAGCTGTGGATTACCCTCCTCAGGCAGATGC-3′ and 5′-GTAATCCACAGCTTTGTTCAC-3′ for ΔPKD3, 5′-ATTGTGCAACCTGAAAACAATAAGCCACCTATAGCCAAGATAACTG-3′ and 5′-CTTATTGTTTTCAGGTTGCACAAT-3′ for ΔPKD4, and 5′-GTCATTGTCAAAGAAGAAATAAACAAAAACCTG GTGGAGATCATCTTG-3′ and 5′-TTTGTTTATTTCTTCTTTGACAATGAC-3′ for ΔPKD5.

To generate the miniAAVR glycosylation mutants, N-linked glycosylation sites in miniAAVR were identified by using UniProt (http://www.uniprot.org/). Five sites were found, namely, N395, N472, N487, N492, and N525. These asparagine residues were mutated to alanine residues in order to prevent N-linked glycosylation from taking place at these sites. Gibson cloning was used to make these mutations. The extreme 5′-end primer (5′-GACTCTAGTCCAGTGTGGTG-3′) and 3′-end primer (5′-ATCCAGAGGTTGATTGTCGAG-3′) were similar for the constructs. The following primers were used to amplify the mutated fragment in the respective constructs: 5′-CATGGGGAAGGCTATGTGGCCGTGACAGTCAAGCCAGAG-3′ and 5′-CTCTGGCTTGACTGTCACGGCCACATAGCCTTCCCCATG-3′ for the N395A mutant, 5′-GTAAACTCGTCCCTGGGGCCTACACTTTCAGCTTGACTGTAG-3′ and 5′-CTACAGTCAAGCTGAAAGTGTAGGCCCCAGGGACGAGTTTAC-3′ for the N472A mutant, 5′-GACTCTGATGGAGCTACCGCCTCTACTACTGCAAACCTGAC-3′ and 5′-GTCAGGTTTGCAGTAGTAGAGGCGGTAGCTCCATCAGAGTC-3′ for the N487A mutant, 5′-GCTACCAACTCTACTACTGCAGCCCTGACAGTGAACAAAGCTGTG-3′ and 5′-CACAGCTTTGTTCACTGTCAGGGCTGCAGTAGTAGAGTTGGTAGC-3′ for the N492A mutant, and 5′-CCATCACCCTCTTTGGGGCCCAGAGCACTGATGATCATGG-3′ and 5′-CCATGATCATCAGTGCTCTGGGCCCCAAAGAGGGTGATGG-3′ for the N525A mutant.

To generate the swap-in constructs of KIAA0319 and AAVR, the following primers were used: 5′-GACTCTAGTCCAGTGTGGTG-3′ and 5′-GACACTCTCTCCAGCAGATACCGTAAGTTCTTTCACTGTC-3′ for fragment 1, 5′-GTATCTGCTGGAGAGAGTGTC-3′ and 5′-CTTATTGTTTTCAGGTTGCACAAT-3′ for fragment 2, and 5′-ATTGTGCAACCTGAAAACAATAAGCCTCCAGTGGCTGTGG-3′ and 5′-ATCCAGAGGTTGATTGTCGAG-3′ for fragment 3 for PKD1-3 AAVR swap-in mutant a; 5′-GGTGGAATTCTGCAGATACCATGGCGCCCCCCACAGG-3′ and 5′-CAATAGCAATGGGGGGCCGATTGACTCTTCTGGCAGGCTTAACAGTGAC-3′ for fragment 1, 5′-ATCGGCCCCCCATTGCTATTG-3′ and 5′-CTGACAGTGAACAAAGCTGTGGA-3′ for fragment 2, and 5′-GACAGTGAACAAAGCTGTGGATTACCCACCAGTTGCTAATGC-3′ and 5′-CGGCCGCCACTGTGCTGGATTTACTTATCGTCGTCATCCTTG-3′ for fragment 3 for PKD2 AAVR swap-in mutant b; 5′-GGTGGAATTCTGCAGATACCATGGCGCCCCCCACAGG-3′ and 5′-CAATAGCAATGGGGGGCCGATTGACTCTTCTGGCAGGCTTAACAGTGAC-3′ for fragment 1, 5′-ATCGGCCCCCCATTGCTATTG-3′ and 5′-TTTATCATCATCAGTGCTTTGACTGCC-3′ for fragment 2, and 5′-GTCAAAGCACTGATGATGATAAAATAGTGAGTTATCATTGGGAAG-3′ and 5′-CGGCCGCCACTGTGCTGGATTTACTTATCGTCGTCATCCTTG-3′ for fragment 3 for PKD2 AAVR swap-in mutant c (aa −4 to 30); 5′-GGTGGAATTCTGCAGATACCATGGCGCCCCCCACAGG-3′ and 5′-CTTCCCAATGGTACTGAACGATTTCAGTATCATCTGTACTTTG-3′ for fragment 1, 5′-ATCGTTCAGTACCATGGGAAG-3′ and 5′-GAGTTTACTTAGTTTTAATATGGCTGTA-3′ for fragment 2, and 5′-CATATTAAAACTAAGTAAACTCGATCCTGGTAACTATAGTTTCAG-3′ and 5′-CGGCCGCCACTGTGCTGGATTTACTTATCGTCGTCATCCTTG-3′ for fragment 3 for PKD 2 AAVR swap-in mutant d (aa 31 to 60); 5′-GGTGGAATTCTGCAGATACCATGGCGCCCCCCACAGG-3′ and 5′-GAAAGTGTAGTTCCCAGGGACAAGGTTAGACAAGCGTAAGACGGGAGAG-3′ for fragment 1, 5′-GTCCCTGGGAACTACACTTTCAG-3′ and 5′-AGCTTTGTTCACTGTCAGGTTTG-3′ for fragment 2, and 5′-GGTGGAATTCTGCAGATACCATGGAGAAGAGGCTGGGAGT-3′ and 5′-AACTGCTACAGGTGGCAGGTTCTTACGGGGCTCTGGCTTGAC-3′ for fragment 3 for PKD2 AAVR swap-in mutant e (aa 61 to 90); and 5′-GGTGGAATTCTGCAGATACCATGGAGAAGAGGCTGGGAGT-3′ and 5′-AACTGCTACAGGTGGCAGGTTCTTACGGGGCTCTGGCTTGAC-3′ for fragment 1, 5′-AACCTGCCACCTGTAGCAGTT-3′ and 5′-TTCAGTATCATCTGTACTTTGGCTGCCA-3′ for fragment 2, and 5′-AAAGTACAGATGATACTGAAATCGTTCAGTACCATTGGGAAG-3′ and 5′-CGGCCGCCACTGTGCTGGATTTACTTATCGTCGTCATCCTTGTAATCC-3′ for PKD2 KIAA0319 swap-in mutant f (aa −4 to 30).

### (ii) Bacterial expression constructs.

For GST-fused PKD expression, the pGEX-4T-3 vector (GE Healthcare Bio-Sciences, Pittsburgh, PA) was used to clone various AAVR PKD-encoding regions, which are diagrammed in Fig. 3A and 6A and B, into BamHI/XhoI sites.

For the expression of polyhistidine (His)-tagged PKD1, PKD2, and PKD3, the PET30a(+) vector (Novagen/EMD Millipore, Billerica, MA) was used to clone PKD1-, PKD2-, and PKD3-encoding sequences through the NdeI/XhoI sites. The coding regions of PKD1, PKD2, and PKD3 are diagrammed in Fig. 3A.

### (iii) rAAV vector constructs.

pAV2RepAV5Cap was constructed by replacing the AAV2 VP-encoding sequence with the AAV5 VP-encoding sequence of pAV5RepCap (53) in pAV2RepCap (psK45) (53). pAV2RepAV1Cap and pAV2AV8Cap were described previously (54, 55). pAV2F5tg83luc-CMVmChery (4.6 kb) is an rAAV2 *cis* transfer plasmid containing the AAV2 inverted terminal repeats (ITR), the simian virus 40 (SV40) poly(A)-firefly luciferase gene-F5tg83 promoter (luciferase expression cassette) (49), the cytomegalovirus (CMV) immediate early (IE) promoter-mCherry gene-bGH (bovine growth hormone) poly(A) (mCherry expression cassette), and the AAV2 ITR, in order.

### Protein expression and purification.

PKD-cloned pGEX-4T-3 or pET30 plasmids were transformed in E. coli strains BL21 and BL21(DE3)/pLysS, respectively. GST-fused PKD and His-tagged PKD proteins were expressed by the induction of isopropyl-β-d-1-thiogalactopyranoside (IPTG) at 1 mM (at an optical density [OD] of 0.6). His-tagged PKD1, PKD2, and PKD3 proteins were purified by using Ni-nitrilotriacetic acid (NTA) agarose (Qiagen, Germantown, MD) under native conditions according to the manufacturer's instructions. Purified proteins were separated on an SDS–12% PAGE gel to ensure a purity of >90%.

### Generation of stable cell lines.

Lentiviral transduction was used to create stable cell lines expressing a selected gene of interest under a CMV promoter. By using the Gibson assembly reaction, the respective genes of interest (see the section on the construction of plasmids, above) were inserted into the pLenti-CMV-Puro-DEST vector and used as described previously (52). Lentivirus was produced by using HEK293 cells and was utilized to transduce the respective cell lines overnight. Cells stably expressing the gene of interest were selected by treatment with 1 to 3 μg/ml puromycin over 2 days (InvivoGen).

### Virus overlay assay.

Proteins were separated by SDS-PAGE or 2-D electrophoresis and electrotransferred onto a nitrocellulose or PVDF membrane. The virus overlay assay was performed as previously described (56). Briefly, the membrane was sequentially incubated with Tris-buffered saline–0.1% Tween 20 (TBS-T) buffer with 10% nonfat milk (NFT), purified WT AAV or rAAV at ∼5 × 10^11^ vgc/ml in TBST–2% NFT, an anti-intact AAV2 or AAV5 antibody at a 1:100 dilution in TBST–2% NFT, a horseradish peroxidase (HRP)-conjugated secondary antibody (Jackson ImmunoResearch, Inc., West Grove, PA), and then SuperSignal West Pico chemiluminescent substrate (Thermo-Fisher) for signal development under a Fujifilm LAS 4000 imager (Fujifilm Life Sciences). Images were processed with Multi Gauge version 2.3 software (Fujifilm Life Sciences).

### Immunoblot analysis.

Proteins were separated by SDS-PAGE or 2-D electrophoresis and electrotransferred onto a nitrocellulose or PVDF membrane. Membranes were blocked by incubation with 1× phosphate-buffered saline (PBS) containing 5% NFT for 1 h at room temperature (RT). Membranes were subsequently incubated overnight at 4°C with primary antibodies in blocking buffer. Membranes were then washed 3 times for 5 min by using wash buffer (1× PBS with 0.1% Tween 20) and further incubated with HRP-conjugated secondary antibodies (anti-mouse and anti-rabbit at 1:5,000 in blocking buffer) (GeneTex) for 1 h at RT. After another set of three washes, antibody-bound proteins were visualized on film by using West Pico and Extended Duration chemiluminescence peroxide solutions (Thermo-Scientific, USA).

### rAAV transduction assay.

Cells were seeded at 10,000 cells/well (96-well plate) overnight. They were then infected with scAAV2-CMV-RFP or scAAV5-CMV-GFP at a multiplicity of infection (MOI) of 20,000 viral genomes (vg)/cell, in complete DMEM. Virus infectivity was determined at 48 h postransduction by measuring transgene expression (red fluorescent protein [RFP] or green fluorescent protein [GFP]) by using flow cytometry. All transductions were performed in triplicate. To measure virus transgene expression (RFP or GFP) in all other experiments, cells were trypsinized 48 h after infection, and a BD LSR Fortessa flow cytometer (BD, Franklin Lakes, NJ, USA) was used to detect fluorescent cells. Data were analyzed and assembled by using FlowJo software (TreeStar, Inc., Ashland, OR, USA).

### Virus neutralization assays. (i) Luciferase assay (Fig. 7D).

HeLa cells were seeded into a 96-well plate at 1 × 10^4^ cells per well 1 day prior to transduction. Purified PKD or the GST protein control was mixed with rAAV(Luc/mCherry) in a volume of 100 μl of PBS (pH 7.4) at various concentrations for 30 min at room temperature. At the time of infection, the medium was removed, and 100 μl of the rAAV(Luc/mCherry)-protein mixture was added to the wells of cells (at an MOI of 7,500 vg per cell). At 3.5 h postinfection at 37°C with 5% CO_2_, the inoculum was removed, the cells were washed with DMEM with 10% FCS briefly, and 100 μl of medium was added to each well. The cells were incubated for 48 h, followed by quantification of luciferase expression. Luciferase activity was quantified by using a luciferase assay system kit (catalogue number E1500; Promega, Madison, WI) according to the manufacturer's instructions, with measurements being taken on a Synergy H1 microplate reader (BioTek, Winooski, VT). The construction and purification of the His-tagged PKD1, PKD2, and PKD3 proteins used in this experiment are described above. The maltose binding protein–PKD1-5 fusion protein used as a control was described previously (21).

### (ii) Fluorescence assay (Fig. 7E).

HeLa cells were seeded into 96-well plates at 10,000 cells per well overnight. Purified, soluble His-tagged AAVR PKD domain constructs were introduced to the medium at the specified concentrations. Cells were then transduced with scAAV-CMV-GFP or scAAV5-CMV-GFP at an MOI of 12,000 vg per cell and incubated for 24 h at 37°C. This was followed by trypsinization and measurement of transgene expression by flow cytometry.
